# Socioeconomic status and oppositional defiant disorder in preschoolers: parenting practices and executive functioning as mediating variables

**DOI:** 10.3389/fpsyg.2015.01412

**Published:** 2015-09-24

**Authors:** Roser Granero, Leonie Louwaars, Lourdes Ezpeleta

**Affiliations:** ^1^Unitat d'Epidemiologia i de Diagnòstic en Psicopatologia del Desenvolupament – Grup de Recerca 2014 SGR 312 Generalitat de Catalunya, Universitat Autonoma de BarcelonaBarcelona, Spain; ^2^Departament de Psicobiologia i Metodologia de les Ciències de la Salut, Universitat Autònoma de BarcelonaBarcelona, Spain; ^3^Departament de Psicologia Clínica i de la Salut, Universitat Autonoma de BarcelonaBarcelona, Spain

**Keywords:** executive functioning, ODD, parenting, preschoolers, socioeconomic status, structural equation modeling

## Abstract

**Objectives:** To investigate the mediating mechanisms of oppositional defiant disorder (ODD) in preschoolers through pathways analysis, considering the family socioeconomic status (SES) as the independent variable and the parenting style and the children's executive functioning (EF) as the mediating factors.

**Method:** The sample included 622 three-year-old children from the general population. Multi-informant reports from parents and teachers were analyzed.

**Results:** Structural Equation Modeling showed that the associations between SES, EF, parenting style and ODD levels differed by children's gender: (a) for girls, the association of low SES and high ODD scores was partially mediated by difficulties in EF inhibition, and parenting practices defined by corporal punishment and inconsistent discipline obtained a quasi-significant indirect effect into the association between SES and ODD; (b) for boys, SES and EF (inhibition and emotional control) had a direct effect on ODD with no mediation.

**Conclusion:** SES seems a good indicator to identify children at high-risk for prevention and intervention programs for ODD. Girls with ODD in families of low SES may particularly benefit from parent training practices and training in inhibition control.

## Introduction

Epidemiological research shows that psychiatric disorders, particularly behavioral problems, are quite common in early childhood. The most frequent behavioral disorder in preschoolers is oppositional defiant disorder (ODD), the prevalence of which is around 10% in the United States community samples (Bufferd et al., 2012) and 7% in Spain (Ezpeleta et al., 2014). ODD is conceptualized as the result of the interaction of individual characteristics (such as a high negative affectivity, low effortful control, difficulties in social learning or emotional dysregulation) with environmental adversities (such as dysfunctional parenting style, parental psychopathology, socioeconomic problems or family conflict). The main objective of this work is to value the mediating mechanisms of ODD in the general population of preschool children, through pathways analysis including the family socioeconomic status, the parenting style and the children's executive functioning (EF).

### Socioeconomic status (SES) as a risk factor for ODD

In the group of environmental adversities, lower SES has showed a strong association with children's behavioral problems. Reiss (2013) describes a clear relationship between socioeconomic deprivation and mental health problems in childhood and adolescence, with low household income and low parental education being the strongest predictors. In studies using samples of typically developing preschoolers, strong associations between family SES and child behavioral problems were found, with a possible mediating role of the neighborhood the family is living in (Kalff et al., 2001; Boyle and Lipman, 2002). Davis et al. (2010) found negative associations between SES and parental report of child emotional symptoms, conduct problems, hyperactivity problems, and/or peer relationship problems. They also found teacher-reported hyperactivity and conduct problems to be associated with lower SES, and with low parental education in particular.

### Parental style as a potential mediator for ODD

Into the environmental adversities domain, parenting style has also showed a strong association with children's behavioral problems, especially with ODD. A clear relation between parenting and problem behavior in young children has been reported with, for example, harsh parenting practices, inconsistent parental discipline, and negative parent emotional expressiveness having unique and cumulative effects on child disruptive behavior (Hughes and Ensor, 2006; Duncombe et al., 2012). Examining the role of early parenting behaviors in predicting later ODD symptoms, Harvey and Metcalfe (2012) found a negative association between maternal warmth and ODD symptoms. They also found that paternal laxness predicted ODD symptoms one year later. Tung et al. (2012) found that harsh punishment was positively associated with parent-rated aggressive behavior and ODD symptomatology.

Parenting behavior and SES seems also related to each other. Conger et al. (1994) examined the link between economic stress in family life and adolescent internalizing and externalizing behavioral symptoms. In their model they found for both parents a significant path from economic pressure to parental hostility through marital conflict or parent-adolescent financial conflict. Pinderhughes et al. (2000) found a significant, direct relation between SES and discipline responses. Indirect relations suggested that these SES differences in discipline responses were due to differences in parenting beliefs and levels of stress. Dodge et al. (1994) found that children from families with lower SES are more likely to receive harsh discipline. Furthermore, their mothers are less warm in their behavior toward the children (Dodge et al., 1994).

### Executive functioning as a potential mediator for ODD

EF are the cognitive processes that enable purposeful and goal-directed behavior and flexible, adaptive responses to changes in the environment (Anderson, 2002; Hughes and Ensor, 2008). They include mental processes such as planning, working memory, inhibition of inappropriate responses, flexibility in adaptation to environmental changes, and decision making (Nigg, 2006). The literature shows a systematic relation between EF and problem behavior in typically developing preschoolers (Espy et al., 2011). Studies in clinical samples usually have a mixture of children with attention deficit/hyperactivity disorder (ADHD) and/or ODD. Given the frequent comorbidity between these disorders, it is hard to distinguish differences in executive functioning associated with each diagnostic type. Some studies show deficits in working memory, planning and organizing and inhibition, but when controlled for ADHD these associations disappear for ODD (Thorell and Wåhlstedt, 2006; Schoemaker et al., 2012; Skogan et al., 2014). However, developmental theorists have proposed the distinction between more abstract-cognitive, “cool” and “hot” EF (Zelazo and Müller, 2002). Cold cognition is thought to be associated with executive functions elicited by abstract and deconceptualized tasks (such as motor response inhibition, attention and cognitive flexibility), and the area of the brain that is utilized for these tasks is the dorsolateral prefrontal cortex. Hot cognition refers to the tasks that require the regulation of emotion or motivation (as well as reevaluating the motivational significance of a stimulus), and it is executed by the activation of the ventral and medial areas of the prefrontal cortex. Some studies have related the association of disruptive disorders such as ODD, conduct disorder (CD) and attention-deficit/hyperactivity disorder (ADHD), with deficits in cool abstract-cognitive functions and hot reward-related EF tasks, but it is unclear what the specific association of ODD with these neuropsychological deficits is, independent of ADHD. A recent study in a clinical sample of *N* = 59 children with a history of early-onset ODD problems (Hobson et al., 2011) observed that ODD with no ADHD is associated with deficits in both hot EF performance (particularly with risky decision-making tasks) and cool EF (slower speeds of inhibitory responding). They concluded that these EF deficits specifically related to ODD should be implicated in theories of antisocial behavior development.

Previous research also shows that in typically developing preschoolers, maternal positive parenting behaviors are related to better child performance in tasks assessing working memory, conflict and impulse control and categorization (Bernier et al., 2010) and that these parental influences already start in infancy (Bernier et al., 2012; Kraybill and Bell, 2013). Furthermore, a relationship between EF and SES has been found, indicating an impact of early access to learning resources on the acquisition of these skills (Clark et al., 2013). Rhoades et al. (2011) found effects of family ecological risk profiles and children's earliest environment, both during infancy, on the development of EF 2 years later. They also imply that this link between SES and EF is at least partially explained by associated parenting behaviors.

### Children's gender and ODD

The literature indicates that the aforementioned relationships of ODD with SES, parenting practices and EF may be different according to the child's gender. At a basic epidemiological level, problem behaviors have obtained higher prevalences in boys (Chen, 2010; Ullebø et al., 2012; Searle et al., 2013). This differentiation, however, was not corroborated for ODD at very young ages (Ezpeleta et al., 2014). Children's gender differences related to parenting practices have reported conflicting outcomes. Tung et al. (2012), for instance, found that inconsistent discipline was positively associated with aggressive behavior and rule breaking only for boys. Other studies have suggested that the influence of parenting behaviors on psychological functioning is probably stronger in girls than in boys (Javo et al., 2004). Kim et al. (2005) found associations between overreactive parenting and externalizing behavior in girls, and between lax parenting and externalizing behavior in boys, while for internalizing behavior the associations were inverse. The association between SES and gender also seems more ambiguous. Some researchers have observed evidence of an equal distribution of externalizing behavior in male and female preadolescents. Mrug and Windle (2009) found more children's externalizing behavior in preadolescents living in neighborhoods with higher poverty rates, but the distribution of these externalizing behaviors were equally distributed between boys and girls. However, Henninger and Luze (2013) concluded an effect of time spent in poverty on the occurrence of externalizing behaviors over time only for girls. Kim et al. (2005) also found a different effect depending on the SES level: the association between overreactive parenting and internalizing behavior in boys was specific to families from low socioeconomic levels. For EF, gender differences may depend on the kind of task executed. Several researchers showed that girls outperform boys in inhibition tasks (Kochanska et al., 2000; Olson et al., 2005). Raaijmakers and colleagues found more EF deficits in boys, specifically in inhibition, verbal fluency, working memory, and set shifting. These differences were found irrespective of aggression or attention problems (Raaijmakers et al., 2008).

### Objectives

Although EF, parenting behaviors and SES have been strongly associated with child behavioral problems, particularly with ODD, no studies have analyzed the underlying processes including these factors and their mediating dependencies in predicting ODD symptomatology levels in preschoolers. Structural-causal modeling is required to assess the direct and indirect associations among the variables explaining the early onset and development of ODD in the context of developmental psychopathology. The objective of this study is to investigate the mediating mechanisms of the ODD levels in preschool age through pathways analysis, using a model that includes as potential predictors family SES, parenting behaviors, children's EF and sex. Based on available empirical evidence, it is hypothesized that: (1) low SES will be directly associated with high ODD scores; (2) the association of SES and ODD will also be mediated by parenting style and executive functioning, and parents from low SES families will score higher on negative parenting behaviors (poor monitoring/supervision, inconsistent discipline, and corporal punishment) which in turn will lead to their children scoring higher on EF deficits; and (3) children from families with low SES, children from parents with negative parenting behaviors, and children with deficits in EF score higher on ODD symptomatology. Regarding children's gender, it is expected that this variable may affect the pathways including SES, EF and parenting, but the lack of empirical evidence prevents us from hypothesizing its specific contribution to the structural modeling.

## Methods

### Participants

The data are part of a longitudinal study concerning risk factors of behavioral problems in preschoolers (Ezpeleta et al., 2014). They were collected through a multisampling design, and corresponded to the in-school population of preschoolers P3-grade (age 3 years old) in Barcelona, Spain. A random sample was considered, inviting 2283 families to be included in the study. The participation rate was 58.7%, which is fairly high for community studies, leading to a sample in the first phase of 1341 families. In this phase semi-public schools were significantly more likely to refuse to participate than public ones (*p* < 0.001), and families with high SES participated more than families with low SES (*p* < 0.001). The children were screened for symptoms of ODD by having their parents complete the Strengths and Difficulties Questionnaire for 3- and 4-year-olds (SDQ^3−4^) (Goodman, 1997). Children with mental retardation and/or pervasive developmental disorders, and families with difficulties with Spanish or Catalan, without a primary caregiver who could report about the child, or who were moving to another city within a year were excluded.

In the second phase all children with a positive screen score (indicating high ODD symptomatology, and defined as a raw score above 4 in the SDQ^3−4^ conduct scale or at least one symptom of the ODD symptoms list) (*n* = 522) were invited to participate, and from the children with negative screen score (indicating low symptomatology, for children who did not meet the positive screen criterion) (*n* = 756) a random 30% was asked to continue. In this phase 10.6% of the families invited refused continued participation. The final sample consisted of *N* = 622 preschoolers and their families, of whom 417 had high ODD symptomatology and 205 were controls. No differences were found when participants and refusals were compared by gender (*p* = 0.82) or by type of school (*p* = 0.85).

The data used in this work correspond to this first assessment at preschool P3 grade, when the participating children were approximately 3 years of age. Final sample included *N* = 604 children with data available in all the measures of the study. No statistical differences emerged on comparing children with all the measures available (*n* = 604) and with missing values in the measures of the study (*n* = 18) in gender (*p* = 0.64), SES (*p* = 0.83) or type of school (*p* = 0.45).

### Instruments

The *Strengths and Difficulties Questionnaire*^3−4^ (SDQ^3−4^) (Goodman, 1997), in its parental version, measured children's psychopathology. This is a brief screening questionnaire with 25 items codified through a 3-point ordered scale (0 = *not true*, 1 = *sometimes true*, and 2 = *certainly true*). The items are factorized in 5 scales: hyperactivity, emotional symptoms, conduct problems, peer problems and prosocial behavior. The conduct problems scale includes four items that correspond to the DSM-IV ODD symptoms “often has temper tantrums or hot tempers,” “generally disobedient, usually does not do what adults request,” “often argumentative with adults,” “can be spiteful to others.” Four additional items were included in the Spanish version to measure the remaining DSM-IV ODD symptoms: “often deliberately annoys others,” “often blames others for his/her mistakes or bad behavior,” “is easily offended by things others say” and “is often angry and resentful.” These eight items allowed the definition of an additional scale, labeled ODD in this work, which measured the DSM-IV ODD symptomatology level. The scores analyzed in this work corresponded to the ODD and hyperactivity (labeled ADHD next) scales, which achieved moderate internal consistency (alpha equal to α = 0.74 and α = 0.67).

The *Alabama Parenting Questionnaire* (APQ) (Frick, Unpublished Rating Scale) measured parenting style through 42 items reported by the parents themselves and grouped into 5 scales: parental involvement, positive parenting, poor monitoring/supervision, inconsistent discipline and corporal punishment. Items are codified in a 5-point Likert scale (1 = *never* to 5 = *always*). The Spanish version of this questionnaire, adapted and validated for preschoolers by de la Osa et al. (2014) in the same sample of this study obtained adequate psychometric evidence (moderate to high internal consistency and good associations with external measures of psychopathology). In this work, the APQ was answered by 604 participating children.

The *Behavior Rating Inventory of Executive Functioning–Preschool version* (BRIEF-P) (Gioia et al., 2003) measured EF reported by children's teachers. It consists of 63 items referring to behaviors with a 3-point ordered scale (0 = *never*, 1 = *sometimes* and 2 = *very often/always*). Items are structured in five clinical scales (the higher the score in each scale, the higher the level of impairment in the construct): inhibition (assessing problems in inhibitory control), shift (difficulties in moving freely among situations, activities, or aspects of a problem), emotional control (difficulties for controlling emotional response), working memory (difficulties in holding information in mind for completing a task), and plan/organize (problems for anticipating future events and taking appropriate steps, and for tapping information to achieve a goal) (Gioia et al., 2003). The Spanish version of the BRIEF-P used in this work achieved adequate psychometric properties (Ezpeleta et al., 2015) in the study's sample itself: high internal consistency (α ≥ 0.87) and moderate convergent validity with other measures of psychopathology and temperament. A total of 94 teachers (96.8% females) from 54 schools completed the BRIEF-P for 620 participating children. Participating teachers had known their students for a mean of 7.6 months (*SD* = 2.2).

The *Hollingshead's Four Factor Index of Social Status* (Hollingshead, 1975) was used to measure families' socioeconomic status. This index corresponds to a family's composite score based on the parents' occupational level (rated on a 9-point scale, in which 9 = higher executive, proprietor of large businesses or major professional and 1 = farm laborers, menial service workers, students or housewives) and educational level (rated on a 7-point scale, in which 7 = graduate/professional training and 1 = less than primary school). The composite family index is computed by multiplying the occupational code by a weight of 5 and the educational code by a weight of 3, and summing the products. Hollingshead Index raw scores range from 8 to 66 (with higher scores reflecting higher SES) and allows placing the families in one of five social classes: 1 = High, 2 = Medium-High, 3 = Medium, 4 = Medium-Low, and 5 = Low. The numbers labeling these five social classes (1–5) represent a range of classes from high to low. For example, a medium-class family has a primary breadwinner who has completed college and could be employed as a school teacher or as an insurance salesperson.

### Procedure

The longitudinal project was approved by the ethics review committee of the institution to which the original authors belonged. The heads of the participating schools, as well as the children's parents, received a complete description of the study. All parents of children from P3-grade were invited to participate and required to complete the screening questionnaire. Families who agreed to be included into the longitudinal study and who met the screening criteria were contacted by telephone and gave written consent. A diagnostic interview with the parents took place at the school by previously trained interviewers who were blind to the children's screening group. After the interview, the parents completed the APQ and were asked about demographic characteristics. The children's teachers were asked to complete the BRIEF-P before the end of the academic year. All the measures for the current study were completed during the P3-grade academic year, when the children were around 3 years old.

### Statistical analysis

Data was analyzed with Stata13 for Windows. Due to the double phase sampling, all the analyses included sample weights to correct for the unequal probabilities of selection: each child was weighted with the reciprocal of its probability of selection in the second phase of the sampling (this allows the generalization of the results to the original general population).

Structural equation modeling (SEM) was conducted to test the hypothesized mediation model that specifies the relationship between SES, parenting behaviors, EF measures and ODD symptoms level. Children's gender was defined as a group variable, since it was expected that boys and girls could obtain different structural coefficients in the pathway. The Maximum Likelihood method of parameter estimation was used and goodness-of-fit was evaluated using the usual statistics: the chi-square test (χ^2^), the Root Mean Square Error of Approximation (RMSEA), the Bentler's comparative Fit Index (CFI), the Tucker-Lewis Index (TLI), and the Standardized Root Mean Square Residual (SRMR). Adequate model fit was considered for non-significant χ^2^ test, RMSEA < 0.08, TLI > 0.9, CFI > 0.9 and SRMR < 0.1. The global predictive capacity of the model was measured with the Coefficient of Determination (CD).

According to the procedures defined by Baron and Kenny (1986) and Sobel (1987), the mediational pathway is considered to occur when the following criteria are met: (a) the independent variable (IV) is statistically associated with the dependent variable (DV); (b) the IV is statistically associated with the hypothesized mediator variable (MV); (c) the hypothesized MV is statistically associated with the DV; and (d) the effect of the IV on the DV diminishes on the addition of the MV to the model. In this study, the mediation test was carried out through Sobel's method (“*estat teffects*” command in Stata13), which breaks total effects down into direct and indirect effects.

Due to the strong comorbid association between ODD and ADHD symptom levels, and the common relationships of both disorders with the variables analyzed in the study, the SDQ-ADHD score was included as a covariate in the SEM.

## Results

### Description of the sample

The mean age of the children analyzed in the study was 3.77 years old (*SD* = 0.33), 302 participants (50%) were boys, 586 (97.0%) were born in Spain and 539 (89.2%) were Caucasian-European (6.3% were Hispanic from South-America and 4.5% pertained to other ethnic groups). Twenty-six children (4.3%) lived in a single parent family and 9 in a reconstructed family (1.5%).

The first rows of Table 1 contains the distribution of the quantitative variables analyzed in this work (range, mean, and standard deviation) for the total sample and stratified by the children's gender. Differences between boys and girls emerged for the mean comparison of BRIEF inhibit, working memory and plan-organize scales, and also for the APQ monitoring scale. The final part of Table 1 contains the distribution of the SES, also for the total sample and for the cohorts defined by children's gender (boys and girls did not differ in the SES levels).

**Table 1:** **Distribution of the variables of the study**.

	**Total sample (*N* = 604)**				**Girls (*N* = 302)**				**Boys (*N* = 302)**				***t_df_*_= 602_**	***p***
	**Min**	**Max**	**Mean**	**SD**	**Min**	**Max**	**Mean**	**SD**	**Min**	**Max**	**Mean**	**SD**		
SDQ-ODD score	0	9	3.8	2.12	0	9	3.7	2.12	0	9	3.8	2.13	0.73	0.469
BRIEF-Inhibit	16	48	23.0	6.91	16	42	21.6	6.20	16	48	24.4	7.30	5.00	<0.001
BRIEF-Shift	10	29	13.2	3.51	10	25	12.9	3.40	10	29	13.4	3.61	1.85	0.064
BRIEF-Emot.Control	9	27	12.1	3.52	9	24	12.0	3.39	9	27	12.3	3.64	0.97	0.333
BRIEF-Working.Mem	17	48	22.5	6.77	17	47	21.4	6.07	17	48	23.7	7.21	4.22	<0.001
BRIEF-Plan/organize	10	30	13.4	3.79	10	27	12.6	3.18	10	30	14.1	4.18	4.95	<0.001
APQ-Positive	5	24	18.4	2.96	10	24	18.5	3.04	5	24	18.3	2.88	0.92	0.358
APQ-Involvement	7	32	20.5	5.83	8	32	20.8	5.79	7	32	20.2	5.85	1.23	0.219
APQ-Monitoring	5	22	12.8	2.02	6	19	12.5	1.93	5	22	13.1	2.07	3.35	0.001
APQ-Corporal	0	9	0.98	1.13	0	5	0.9	1.00	0	9	1.05	1.24	1.60	0.110
APQ-Inconsistent	0	15	4.17	2.33	0	15	4.23	2.25	0	13	4.11	2.41	0.59	0.554
SDQ-ADHD *covariate*	0	10	3.89	2.49	0	10	3.71	2.52	0	10	4.07	2.45	1.74	0.083
	***n***	**%**			***n***	**%**			***n***	**%**			**χ^2^_*df* = 4_**	***p***
SES High	199	32.9			98	32.5			101	33.4			1.57	0.813
Medium-high	188	31.1			93	30.8			95	31.5				
Medium	86	14.2			40	13.2			46	15.2				
Medium-low	96	15.9			51	16.9			45	14.9				
Low	35	5.8			20	6.6			15	5.0				

BRIEF, Behavior Rating Inventory of Executive Functioning–Preschool version; APQ, Alabama Parenting Questionnaire; SDQ, Strengths and Difficulties Questionnaire; SD, Standard deviation; SES, Socioeconomic Status.

### Structural equation model

Table 2 shows the correlation-matrix for the variables of the study. Since one of the objectives of this work is to value the potential differences in the pathways due to sex, correlation matrixes have been computed stratified by children's gender. The APQ and BRIEF-P scores which achieved the strongest associations with the SES and/or the ODD levels (in any of the correlation matrices, for boys and/or girls) were considered for an initial SEM: (a) EF factors of inhibit, emotional control, working memory and plan-organize; and (b) parental style dimensions of corporal punishment, inconsistent discipline, and involvement. During the modeling process, the BRIE-P working memory and plan-organize scores were excluded since these measures obtained high correlations with the other two BRIEF-P scales considered for the SEM and entailed worse or lack of fitting. The APQ involvement scale score was also excluded of the final SEM because it worsened goodness-of-fit.

**Table 2:** **Correlations between the variables of the study**.

		**2**	**3**	**4**	**5**	**6**	**7**	**8**	**9**	**10**	**11**	**12**	**13**
**Girls**													
1	SDQ-ODD score	0.17^*^	−0.10	0.10	0.11	0.14^*^	−0.07	−0.18^*^	0.03	0.18^*^	0.22^*^	0.20^*^	0.28^*^
2	BRIEF-Inhibit		0.24^*^	0.54^*^	0.59^*^	0.57^*^	0.06	−0.01	0.02	0.03	0.07	0.17^*^	0.27^*^
3	BRIEF-Shift			0.56^*^	0.35^*^	0.37^*^	0.01	0.05	0.02	−0.05	−0.05	−0.02	−0.06
4	BRIEF-Emotional Control				0.42^*^	0.42^*^	−0.04	−0.07	−0.03	0.04	0.04	0.01	0.01
5	BRIEF-Working memory					0.89^*^	0.13^*^	0.05	−0.04	0.03	0.03	0.16^*^	0.18^*^
6	BRIEF-Plan/organize					.11	0.01	−0.04	0.02	0.02	0.09	0.14^*^
7	APQ-Positive							0.72^*^	0.11	−0.08	−0.35^*^	0.09	0.05
8	APQ-Involvement								0.14^*^	−0.12^*^	−0.39^*^	−0.10	−0.04
9	APQ-Monitoring									0.06	0.08	0.10	0.04
10	APQ-Corporal										0.18^*^	0.14^*^	0.12^*^
11	APQ-Inconsistent											0.14^*^	0.13^*^
12	Socioeconomic status												0.33^*^
13	SDQ-ADHD *(covariate)*												
**BOYS**													
1	SDQ-ODD score	0.21^*^	0.09	0.20^*^	0.21^*^	0.19^*^	−0.09	−0.13^*^	0.10	0.10	0.10	0.15^*^	0.31^*^
2	BRIEF-Inhibit		0.21^*^	0.54^*^	0.65^*^	0.61^*^	0.02	−0.05	0.10	0.08	−0.03	0.01	0.38^*^
3	BRIEF-Shift			0.55^*^	0.42^*^	0.46^*^	0.14^*^	0.03	0.18^*^	−0.02	0.00	0.06	−0.04
4	BRIEF-Emotional Control				0.41^*^	0.44^*^	0.06	−0.06	0.11	0.06	−0.08	−0.01	0.13^*^
5	BRIEF-Working memory					0.90^*^	0.09	−0.03	0.10	0.03	0.02	0.11	0.34^*^
6	BRIEF-Plan/organize						0.08	−0.03	0.11	0.08	0.04	0.06	0.30^*^
7	APQ-Positive							0.67^*^	0.06	−0.01	−0.27^*^	0.02	−0.05
8	APQ-Involvement								−0.08	−0.19^*^	−0.36^*^	−0.18^*^	−0.13^*^
9	APQ-Monitoring									0.17^*^	0.22^*^	0.12^*^	0.10
10	APQ-Corporal										0.11	0.05	0.04
11	APQ-Inconsistent											0.12^*^	0.16^*^
12	Socioeconomic status												0.19^*^
13	SDQ-ADHD *(covariate)*												

BRIEF, Behavior Rating Inventory of Executive Functioning–Preschool version; SES, Socioeconomic Status; APQ, Alabama Parenting Questionnaire; SDQ, Strengths and Difficulties Questionnaire.

* Significant correlation (0.05 level).

**Figure 1:**
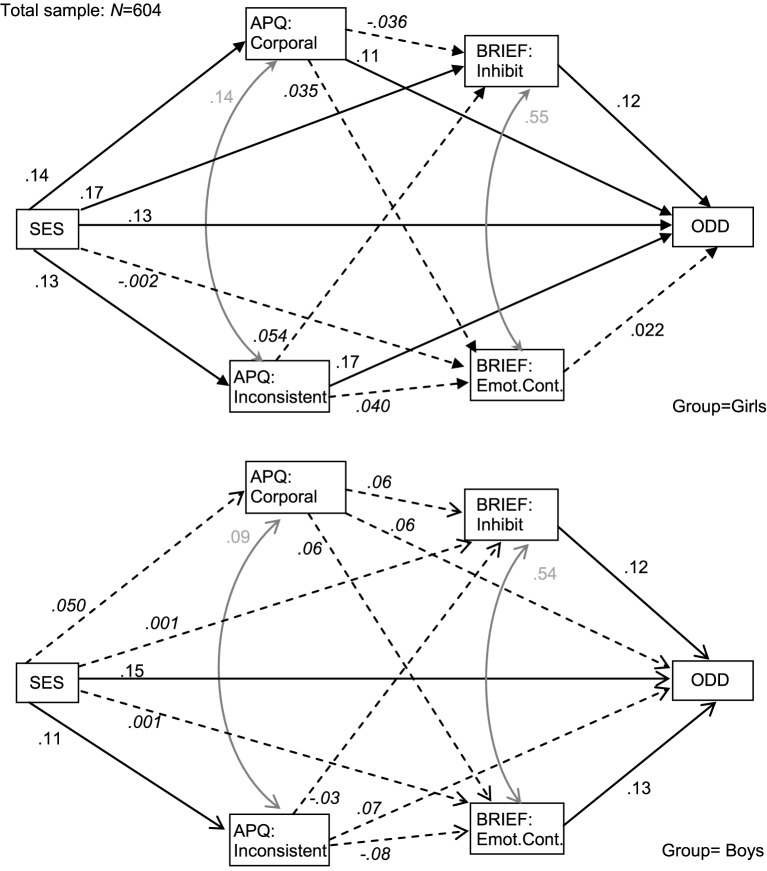
**SEM (adjusted by SDQ-ADHD score) including children's gender as group variable**. Discontinued-line and italics: non-significant associations. Attenuated line: inter-correlations between variables. SES, Socioeconomic Status; APQ, Alabama Parenting Questionnaire; BRIEF, Behavior Rating Inventory of Executive Functioning–Preschool version; SDQ, Strengths and Difficulties Questionnaire.

Figure 1 shows the final SEM measuring the underlying process between SES, EF, parenting style and ODD at age 3, adjusted by SDQ^3−4^-ADHD score (Table 3 details the structural coefficients). Goodness-of-fit of the new model was achieved: χ^2^ = 1.835 (*p* = 0.399), RMSEA = 0.001, CFI = 0.999, TLI = 0.999, and SRMR = 0.012. To assess the potential differences in the SEM due to gender, measurement invariance across the groups has been tested by comparing the previous unconstrained model with a new model in which loadings and intercepts were constrained to be equal across boys and girls. Since the χ^2^ difference statistic reveals a significant difference between models (χ^2^ = 54.02, *p* < 0.001), a lack of invariance across gender is assumed.

**Table 3:** **Structural coefficients of the final SEM diagram (*N* = 604)**.

			**Coefficient**	**SE**	***z***	***p***	**95% CI (coefficient)**	
APQ_Corporal	Socioecon.Status	*Girls*	0.1347	0.0604	2.23	0.026	0.0164	0.2530
		*Boys*	0.0499	0.0540	0.92	0.355	−0.0559	0.1557
APQ_Inconsistent	Socioecon.Status	*Girls*	0.1309	0.0642	2.04	0.042	0.0050	0.2568
		*Boys*	0.1096	0.0602	1.82	0.049	0.0083	0.2275
BRIEF_Inhibit	APQ_ Corporal	*Girls*	−0.0358	0.0549	−0.65	0.515	−0.1433	0.0718
		*Boys*	0.0639	0.0483	1.32	0.186	−0.0308	0.1587
	APQ_Inconsistent	*Girls*	0.0542	0.0481	1.13	0.260	−0.0400	0.1485
		*Boys*	−0.0310	0.0581	−0.53	0.594	−0.1449	0.0829
	Socioecon.Status	*Girls*	0.1762	0.0615	2.86	0.004	0.0556	0.2968
		*Boys*	0.0006	0.0606	0.01	0.992	−0.1183	0.1194
BRIEF_EmotControl	APQ_Corporal	*Girls*	0.0351	0.0623	0.56	0.573	−0.0870	0.1571
		*Boys*	0.0621	0.0561	1.11	0.268	−0.0478	0.1720
	APQ_Inconsistent	*Girls*	0.0402	0.0544	0.74	0.461	−0.0665	0.1469
		*Boys*	−0.0891	0.0573	−1.55	0.120	−0.2014	0.0232
	Socioecon.Status	*Girls*	−0.0018	0.0629	−0.03	0.978	−0.1251	0.1216
		*Boys*	0.0013	0.0628	0.02	0.984	−0.1218	0.1243
SDQ_ODD	APQ_Corporal	*Girls*	0.1128	0.0542	2.08	0.037	0.0066	0.2190
		*Boys*	0.0553	0.0407	1.36	0.174	−0.0244	0.1350
	APQ_Inconsistent	*Girls*	0.1658	0.0600	2.76	0.006	0.0481	0.2835
		*Boys*	0.0710	0.0503	1.41	0.158	−0.0276	0.1697
	BRIEF_Inhibit	*Girls*	0.1246	0.0658	1.89	0.058	−0.0045	0.2536
		*Boys*	0.1225	0.0715	1.71	0.047	0.0176	0.2626
	BRIEF_EmotCont	*Girls*	0.0225	0.0601	0.37	0.708	−0.0953	0.1403
		*Boys*	0.1343	0.0724	1.85	0.044	0.0076	0.2761
	Socioecon.Status	*Girls*	0.1302	0.0540	2.41	0.016	0.0244	0.2360
		*Boys*	0.1488	0.0544	2.74	0.006	0.0423	0.2554

SES, Socioeconomic Status; APQ, Alabama Parenting Questionnaire; BRIEF, Behavior Rating Inventory of Executive Functioning–Preschool version; SDQ, Strengths and Difficulties Questionnaire.

The pathway diagram for girls showed that high ODD level was directly predicted by lower SES levels, high scores in the parenting style variables of corporal punishment and inconsistence, and high scores in the EF measure of inhibition. Parenting style measures were potential partial mediator variables between SES and ODD: lower SES predicted higher scores in corporal punishment and inconsistence, and higher scores in both parenting scales were related to higher ODD level. The BRIEF-P-inhibit score was also a potential partial mediating factor in the pathway between SES and ODD: lower SES related to greater impairments in inhibition and higher impaired inhibition related to higher ODD level. No statistical association was found between SES and the BRIEF-P-emotional control score, nor between emotional control and ODD level. EF measures contemplated in the pathway were not mediating factors between parenting style and ODD level. The global predictive capacity of the girls' structural pathway model was *CD* = 0.15.

Structural coefficients for boys showed few statistical associations. Higher ODD levels were predicted by lower SES and higher scores on the EF inhibition and emotional control scales. Parenting style measured by corporal punishment and inconsistence dimensions did not influence EF or ODD levels. The global predictive capacity of the boys' structural model was lower than for girls (*CD* = 0.06).

Table 4 contains the global mediation tests for the complete pathway showed in Figure 1 differentiating between direct and indirect effects for the dependent variable ODD level. Results show that the socioeconomic status achieved a significant direct effect (*z* = 2.40, *p* = 0.017) and also a significant indirect effect (*z* = 2.60, *p* = 0.009) on the ODD score for the girls' pathway, which suggest that the relationship between SES and ODD level is partially mediated by parenting and/or EF. To identify what were the specific mediators of the ODD levels, individual-specific mediation tests were carried into the girl's model. Results showed that EF variables (inhibit and emotional control dimensions) did not mediated into the relationships between the parenting style variables (corporal punishment and inconsistent discipline) and ODD (that is, non-mediation mechanism was found for the paths APQ->BRIEF->ODD). However, the parenting style variables obtained significant direct effects (punishment: *z* = 2.95, *p* = 0.003; inconsistence: *z* = 3.14, *p* = 0.002) and quasi-significant indirect effects (punishment: *z* = 1.74, *p* = 0.082; inconsistence: *z* = 1.75, *p* = 0.080) into the relationships between SES->APQ->ODD. The BRIEF inhibit score also achieved a significant direct effect (*z* = 2.66, *p* = 0.008) and a significant indirect effect (*z* = 1.96, *p* = 0.050) into the relationship between SES->BRIEF->ODD.

**Table 4:** **Sobel mediation tests for the global SEM: direct and indirect effects of APQ and BRIEF on the dependent variable ODD level**.

**Global tests**	**Direct effects**						**Indirect effects**						**Total effects**					
	**Coefficient**	**SE**	***z***	***p***	**95%CI**		**Coefficient**	**SE**	***z***	***p***	**95%CI**		**Coefficient**	**SE**	***z***	***p***	**95%CI**	
**APQ-corporal**																		
Girls	0.246	0.117	2.11	0.035	0.018	0.475	−0.008	0.017	−0.48	0.629	−0.040	0.024	0.238	0.116	2.05	0.040	0.011	0.465
Boys	0.093	0.069	1.35	0.178	−0.042	0.227	0.027	0.020	1.35	0.176	−0.012	0.066	0.120	0.072	1.66	0.096	−0.021	0.260
**APQ-inconsistent**																		
Girls	0.159	0.056	2.83	0.005	0.049	0.270	0.007	0.006	1.14	0.255	−0.005	0.020	0.167	0.057	2.93	0.003	0.055	0.279
Boys	0.062	0.044	1.42	0.156	−0.024	0.149	−0.014	0.011	−1.22	0.221	−0.036	0.008	0.049	0.045	1.08	0.280	−0.0040	0.137
**BRIEF-inhibit**																		
Girls	0.042	0.022	1.91	0.056	−0.001	0.085	^*^	^*^	^*^	^*^	^*^	^*^	0.042	0.022	1.91	0.058	−0.001	0.085
Boys	0.035	0.021	1.70	0.089	−0.005	0.076	^*^	^*^	^*^	^*^	^*^	^*^	0.035	0.021	1.70	0.047	0.005	0.076
**BRIEF-emot.cont**.																		
Girls	0.014	0.038	0.38	0.708	−0.060	0.089	^*^	^*^	^*^	^*^	^*^	^*^	0.014	0.038	0.38	0.708	−0.060	0.089
Boys	0.078	0.042	1.85	0.065	−0.005	0.160	^*^	^*^	^*^	^*^	^*^	^*^	0.078	0.042	1.85	0.044	0.005	0.160
**SES**																		
Girls	0.232	0.097	2.40	0.017	0.042	0.422	0.106	0.041	2.60	0.009	0.026	0.186	0.338	0.099	3.40	0.001	0.143	0.533
Boys	0.262	0.098	2.67	0.008	0.070	0.455	0.017	0.029	0.59	0.553	−0.040	0.075	0.280	0.104	2.70	0.007	0.077	0.483

* Not applicable.

## Discussion

Final SEM analyses showed that high ODD symptom levels were predicted by low SES levels, high scores in parenting behaviors characterized by corporal punishment and inconsistent discipline, and high scores in EF inhibit and emotional control dimensions. Children's gender differences emerged in the pathway: (a) the ODD level score for boys was directly predicted by SES and EF inhibit and emotional control dimensions, while parenting scores did not obtain significant associations with any other variables analyzed; (b) the ODD level score for girls was directly predicted by SES, parenting practices (corporal punishment and inconsistent scores) and EF inhibit scale, the parenting practices analyzed obtained quasi-significant indirect effects into the relationship between SES and ODD, and EF inhibit dimension achieved a significant indirect effect into the relationship between SES and ODD. These results suggest that parenting style (defined by the dimensions corporal punishment and consistence) and EF inhibit level should achieve a partial mediation role between SES and ODD for preschooler girls from the general population.

The direct association between lower SES and ODD symptomatology is congruent with the literature (Brooks-Gunn and Duncan, 1997; Mistry et al., 2002). In the final SEM model of this work, boys are slightly more sensitive to the effect of lower SES, showing no influence of parenting practices, often associated with SES. This may be due to a combination of boys showing more externalizing behavior in general (Miner and Clarke-Stewart, 2008; Searle et al., 2013) and parents with low SES perceiving their children exhibiting more at-risk levels of aggression than parents with higher SES (Graves et al., 2012). One might suspect that boys from lower SES families would be at greater risk of exhibiting externalizing behavior than girls from lower SES families or boys from higher SES families. However, no significant difference was found between ODD scores for boys and girls in our study, and correlations did not suggest a stronger association between SES and ODD scores for boys.

For girls the association between SES and ODD is more complex than for boys, with both negative parenting behaviors and EF deficits acting as separate partially mediators. Lower SES is associated with more corporal punishment and more use of inconsistent discipline, which in turn are both related to higher levels of ODD symptoms. Several studies have reported less maternal warmth and more harsh discipline in families with low SES (Pinderhughes et al., 2000; Callahan and Eyberg, 2010), and this association has been corroborated in our study for boys considering the inconsistency score. For girls, lower SES was associated not only with inconsistent discipline but also with corporal punishment. A possible explanation for this gender difference could be that boys, based on gender stereotypes, are more likely to receive harsh physical discipline in general (Mahoney et al., 2000), regardless of ODD. Parents may still believe that boys need more physical discipline in order to change their behavior than girls do (McKee et al., 2007). Therefore, it would be less remarkable that boys in lower SES families experience harsh discipline: boys in families with higher SES experience the same amount. Girls in lower SES families however, endure more harsh discipline than girls from high SES families, resulting in a clear association between lower SES and more corporal punishment. In our study, the mean APQ-corporal score was statistically higher for girls in low SES level compared to girls in high SES (1.19 vs. 0.84, *p* = 0.016), and also for boys compared to girls when the comparison was carried out in higher SES levels (means equal to 1.06 vs. 0.84, *p* = 0.033).

Besides a consistent relationship between lower SES and negative parenting practices, harsh parenting practices have been found to have a unique effect on child disruptive behavior, even in early childhood (Duncombe et al., 2012; Harvey and Metcalfe, 2012). Although the association is not clear in the literature, some studies confirm a greater influence of parenting practices on problem behavior in girls (Javo et al., 2004). Kim et al. (2005) found that girls' externalizing behavior is associated with overreactive parenting, whereas for boys this relation did not occur in their study. The authors attributed this to gender stereotypes as well. With aggressive behavior more acceptable in boys than in girls and shy and dependent behavior more acceptable in girls than in boys, Kim and colleagues offer the possibility that parents tend to be overreactive to behaviors that are incongruent with these gender stereotypes by punishing stereotype-inconsistent behavior.

The association between SES and ODD in girls was also partially mediated by the EF aspect inhibition, given the associations between lower SES and deficits in inhibition and between deficits in inhibition and ODD symptoms. The relation between SES and EF seems clear in the literature, with more EF deficits in children from lower SES families (Rhoades et al., 2011; Clark et al., 2013). The associations between EF inhibit and ODD symptoms in the SEM model are remarkable though, for both boys and girls, since the model is controlled for ADHD. Several studies have found relations between EF and ODD, but as soon as ADHD was taken into account, the associations disappeared (Thorell and Wåhlstedt, 2006; Schoemaker et al., 2012). For girls, inhibition acted as a partial mediator in the association between SES and ODD symptoms. As mentioned before, girls tend to perform better on inhibition tasks than boys (Kochanska et al., 2000; Olson et al., 2005). In our study girls scored significantly lower than boys on BRIEF-Inhibit (21.62 vs. 24.40, *p* < 0.001), indicating fewer deficits in inhibition. Especially in early childhood this could have to do with girls maturing faster than boys. They develop some self-regulatory and communicative skills earlier than boys and are therefore better in regulating their emotions and behavior (Keenan and Shaw, 1997; Bornstein et al., 2008). With these abilities developed to a further stage than boys at the same age, they develop more positive relationships with peers and parents, communicate their needs more effectively and their needs are therefore met more often. This in turn results in less frustration and fewer conflicts and thus less externalizing behavior (Keenan and Shaw, 1997). In contrast, girls who show deficits in the development of inhibitory and regulatory abilities miss the communication advantages and may therefore exhibit more externalizing behavior.

The role of EF emotional control in the SEM models is relevant. Emotional control is considered as a hot EF task, which may be more associated with ODD than cool EF (Zelazo and Müller, 2002). Finding this relationship in the model for boys supports the hypothesized association between hot EF and ODD. Research on this topic is scarce in preschoolers, and therefore the current finding is important in the research concerning ODD in young children. However, it is present in boys only, and not in girls.

The strengths of this work include a large community sample of young preschoolers (3 years old), the analysis of ODD differentiated from ADHD (many studies combine ODD + ADHD, making it hard to distinguish between findings for the different disorders) and the modeling through SEM to obtain a *causal* model of the underlying process of ODD.

The main limitations of the study include: (a) the cross-sectional nature of the data, which formally prevents causal interpretations of the pathways analyses in light of potential common reciprocal associations between ODD and parenting; (b) the analysis of data gathered only through questionnaire (the BRIEF-P answered by the teachers and the APQ answered by parents); and (c) the limited range of families' SES (particularly from low SES categories).

This study contributes some insights which may be helpful for understanding the mechanisms by which SES affects the development of ODD and in developing interventions for young children at risk for ODD. The study suggests that SES is a good indicator for selecting children at high risk and it confirms the need to involve parents in preventive and intervention programs. Parents can adjust their behavior toward their child in a more constructive way and perhaps prevent their child's behavior from escalating or help to turn it around. Parental involvement seems particularly important for girls. Another important insight of this study is that intervention programs for boys and girls may have different components. Whereas for girls an important focus should be on parental involvement and the development of inhibitory abilities, for boys programs should focus on the development of emotional control as well.

Future studies are required in order to increase the knowledge of the underlying process of ODD during preschool age, including new constructs (and their reciprocal relationships) and multi-informant measures. Developmental differences should also be considered in future pathways analyses, to assess if the underlying processes between SES, parenting, EF and ODD could be dependent on children's age. In addition, due to the strong association between ODD, CD, and ADHD, it should be also relevant to obtain empirical evidence of the pathways for the CD and the ADHD levels controlling for the presence of ODD.

## Author contributions

All authors have made substantial contributions to the work reported in the manuscript: LE designed the study and was responsible for assessment instruments. LL conducted literature searches and provided summaries of previous research studies. She also wrote the first draft of the manuscript. RG designed and conducted the statistical analysis. All the authors reviewed, contributed to and have approved the final manuscript.

## Funding sources

Funding for this study was provided by Spanish Ministry of Science and Innovation PSI2009-07542, the Spanish Ministry of Economy and Competiveness PSI2012-32695 and the Secretaria d′Universitats i Recerca del Departament d′Economia i Coneixement de la Generalitat de Catalunya (2014 SGR 312). These funding sources had no role in the study design, collection, analysis or interpretation of the data, writing the manuscript, or the decision to submit the paper for publication. The terms of this arrangement have been reviewed and approved by the Autonomous University of Barcelona in accordance with its policy on research.

## Conflict of interest statement

The authors declare that the research was conducted in the absence of any commercial or financial relationships that could be construed as a potential conflict of interest.
